# Aerial Application of Pheromones for Mating Disruption of an Invasive Moth as a Potential Eradication Tool

**DOI:** 10.1371/journal.pone.0043767

**Published:** 2012-08-24

**Authors:** Eckehard G. Brockerhoff, David M. Suckling, Mark Kimberley, Brian Richardson, Graham Coker, Stefan Gous, Jessica L. Kerr, David M. Cowan, David R. Lance, Tara Strand, Aijun Zhang

**Affiliations:** 1 Scion (New Zealand Forest Research Institute), Christchurch, New Zealand; 2 The New Zealand Institute for Plant & Food Research Ltd, Lincoln, New Zealand; 3 Scion (New Zealand Forest Research Institute), Rotorua, New Zealand; 4 Plant Protection and Quarantine, United States Department of Agriculture, Animal and Plant Health Inspection Service, Buzzards Bay, Massachusetts, United States of America; 5 Pacific Wildland Fire Sciences Laboratory, United States Department of Agriculture, Forest Service, Seattle, Washington, United States of America; 6 Invasive Insect Biocontrol and Behavior Laboratory, United States Department of Agriculture, Agricultural Research Services, Beltsville Agricultural Research Center - West, Beltsville, Maryland, United States of America; United States Department of Agriculture, United States of America

## Abstract

Biological invasions can cause major ecological and economic impacts. During the early stages of invasions, eradication is desirable but tactics are lacking that are both effective and have minimal non-target effects. Mating disruption, which may meet these criteria, was initially chosen to respond to the incursion of light brown apple moth, *Epiphyas postvittana* (LBAM; Lepidoptera: Tortricidae), in California. The large size and limited accessibility of the infested area favored aerial application. Moth sex pheromone formulations for potential use in California or elsewhere were tested in a pine forest in New Zealand where LBAM is abundant. Formulations were applied by helicopter at a target rate of 40 g pheromone per ha. Trap catch before and after application was used to assess the efficacy and longevity of formulations, in comparison with plots treated with ground-applied pheromone dispensers and untreated control plots. Traps placed at different heights showed LBAM was abundant in the upper canopy of tall trees, which complicates control attempts. A wax formulation and polyethylene dispensers were most effective and provided trap shut-down near ground level for 10 weeks. Only the wax formulation was effective in the upper canopy. As the pheromone blend contained a behavioral antagonist for LBAM, ‘false trail following’ could be ruled out as a mechanism explaining trap shutdown. Therefore, ‘sensory impairment’ and ‘masking of females’ are the main modes of operation. Mating disruption enhances Allee effects which contribute to negative growth of small populations and, therefore, it is highly suitable for area-wide control and eradication of biological invaders.

## Introduction

Biological invasions resulting from trade and other human activities represent a major threat to biodiversity and the integrity of ecosystems [1]. The economic impact of invasive species can reach billions of dollars annually, resulting from control costs and loss of commodities as well as loss of ecosystem goods and services and other non-market values [2], [3]. Some invaders, such as emerald ash borer [4] and the pathogens that cause chestnut blight [5] and Dutch elm disease [6], are particularly harmful and can cause large-scale ongoing damage or even the near disappearance of their host plants. Despite our increasing awareness of biosecurity issues and attempts to mitigate invasion pathways, increasing international trade makes the ongoing arrival of exotic species inevitable. For example, establishments of forest insects in the U.S. continue at a rate of ca. 2.5 species per year [7].

Biological invasions and their impacts are mostly irreversible except when invaders are detected early enough, while their distribution is still limited, when area-wide eradication may be feasible. Successful eradications are difficult to achieve although our increasing understanding of the dynamics of invading populations [8] and of the integrated use of tools has led to several successful campaigns against insects, mammals, and plants [9], [10], [11], [12]. Despite these successes, eradications that involve the use of pesticides, which may have non-target effects, face increasing opposition [13]. Mating disruption, the use of synthetic sex pheromones which interfere with mate finding and reproduction, is recognized as an environmentally friendly method for the management of pest insects [14], [15], [16], [17]. Mating disruption (MD) uses small amounts of naturally occurring compounds that function as odors rather than toxins, it is highly target-specific, and can be combined with other integrated pest management techniques. Although MD is now widely used for crop protection over smaller areas, its use for area-wide control and eradication has been limited to few species [16]. MD by aerial application of moth pheromones has been widely used in the U.S. for the gypsy moth, *Lymantria dispar*, “slow-the-spread” program; up to 2008 more than 1.4 million ha were treated with MD formulations [10]. This is considered environmentally friendly and more cost-effective than alternatives for suppressing gypsy moth populations [18]. Pink bollworm control has also relied on pheromone application over large areas integrated with other methods [19]. MD may be particularly successful for the management of recent invaders because it can enhance Allee effects that increase extinction pressure [8].

*Epiphyas postvittana* (Lepidoptera: Tortricidae), the light brown apple moth (LBAM) is an economically important pest, especially in horticulture in its native Australia and in New Zealand where it was introduced over 100 years ago [20]. A detection of the highly polyphagous LBAM in California in 2007 led to a response by federal and state authorities aiming at eradication or containment [20]. LBAM has also become established in Hawaii, the UK and Ireland, proving that it is a successful invader [20]. Although biological control and IPM programs, using insecticides and other methods, contribute to successful management in New Zealand, LBAM remains very abundant in organic orchards [21] and pine forests [22]. However, strict control regimes are required for quarantine reasons as there is zero tolerance on export fruit [23]. California is a major producer and exporter of a wide range of horticultural and other crops, and the establishment and potential spread of LBAM was expected to cause considerable crop damage and impediments to international and domestic trade [24], as well as direct and indirect environmental damage.

Given the scale of the LBAM infestation evident across several hundred square kilometers in 2007, aerial treatment was considered the only effective way of covering such a large area, using pheromone formulations as the most appropriate eradication technology [20]. Use of conventional insecticides in the mostly urban and peri-urban areas was ruled out. MD for LBAM using ground-applied dispensers was first developed in New Zealand in pine plantations and orchards [25]. Today, MD is used with success in Australia and New Zealand for LBAM control in citrus orchards [26] and organic apple orchards [21], [23]. But our experience with aerial application of formulations for MD is limited. In California, two initial aerial applications of microencapsulated LBAM pheromone (using an incomplete blend without a ‘sticker’) were made in 2007 over ca. 20,000 ha in Monterey and Santa Cruz counties [20]. The primary means of assessment of efficacy was disruption of pheromone trap catch, however traps operated within the treated and untreated zones provided unclear results [20]. The use of MD for the incursion response in California has been criticized and its effectiveness has been questioned [27]. Before further consideration of pheromone applications, it was necessary to test the efficacy and longevity of different formulations experimentally in areas where LBAM was abundant.

Here we report the results of a substantial study involving aerial application by helicopter of four different pheromone formulations as well as hand-applied pheromone polyethylene tubing dispensers (hereafter referred to as ‘twist-ties’) in New Zealand, in a pine forest with abundant LBAM [22]. Formulations were tested in 5 ha plots (Fig. S1, Fig. S2), replicated five times. A total of 802 traps baited with either synthetic lures or female moths were used before and after application to assess populations and treatment effects (i.e., whether pheromone trap catch was inhibited, which is indicative of females not being able to attract males for mating). Vertical trap transects were installed from near the ground up to a height of 17 m to monitor the vertical distribution of LBAM and to assess treatment effects in stands of taller trees, in the canopy of trees as well as near ground level. We hypothesized that successful MD was more difficult to achieve in the upper canopy than at ground level because of the lower surface area available for deposition of formulations and dilution of airborne pheromone associated with greater wind speeds. We also examined the longevity of the different formulations by quantifying pheromone evaporation over time, which is expected to affect the resulting aerial concentrations and effectiveness of MD. This study is important not only for the response to the incursion of LBAM in California but also for similar uses against other species where ‘greener’ yet effective alternative incursion response and management methods are required.

## Results

The results are presented in four section addressing (1) mating disruption treatment effects near ground level and relationships with pheromone release characteristics of formulations, (2) effects of horizontal trap position (i.e., edge effects from the center to beyond the treated area), (3) distribution of catches in vertical transects across the canopy, and (4) mating disruption effects at different heights across the canopy.

### LBAM Trap Catches and Treatment Effects Near Ground Level

**Figure 1:**
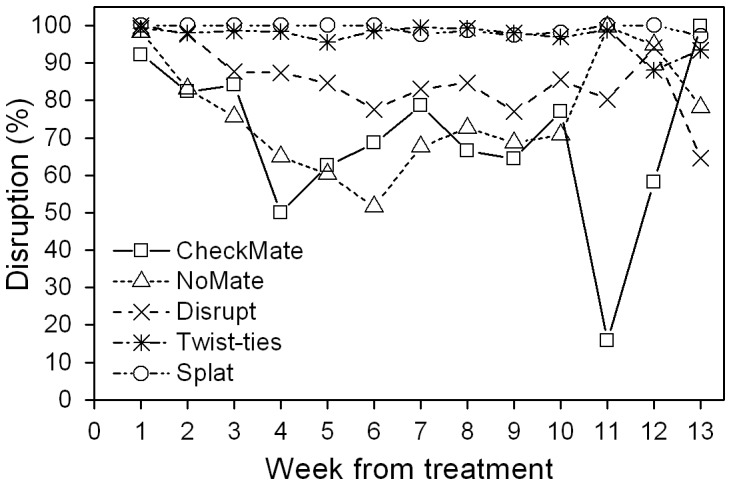
Effects of application of pheromone formulations on trap catch (percent disruption) of light brown apple moth. Note: Percent disruption is the difference in presence between ‘treated’ and ‘controls’ expressed as a percentage of the ‘control’ (values are based on back-transformed covariate-adjusted percent moth presence in traps – see Fig. S3). Results shown are for traps at 1.5 m above ground. Data for weeks 11–13 are based on very low catches at the end of the flight period and percent disruption in those weeks should be interpreted with caution.

Over 28,700 male LBAM were trapped during this trial, of which over 25,000 catches occurred near ground level (i.e., at ca. 1.5 m) where mating disruption is usually assessed, and where the majority of our traps were located. The covariate-adjusted mean catch and percent presence following the application of treatments (log-transformed trap catch for each plot in the 2 weeks prior to treatment was used as a covariate) showed that treatment effects were significant for traps at 1.5 meters across the entire treatment period (see Table S1 for ANCOVA results) except near the end of the flight period when catches generally declined. In the first few weeks following treatment, catches were considerably reduced in all treatments, compared with the controls. All pheromone formulations provided better than 90% disruption in the first week post treatment and better than 80% in the second week (Fig. 1). Traps in several treatments started catching considerable numbers of LBAM from week 5 and catches were recorded in over 10% of the traps in plots treated with CheckMate and NoMate (Fig. S3), providing less than 65% disruption in terms of absence of catches (Fig. 1). Traps in plots treated with Splat and twist-ties remained suppressed, with the Disrupt flakes providing an intermediate effect. Splat and twist-tie treatments maintained between 95% and near 100% disruption during the 10 weeks following application while moth activity was sufficient for evaluation (Fig. 1). Summed over the entire 13 weeks post treatment, all formulations except CheckMate reduced catches significantly from the controls (Table 1). Plots treated with Splat and twist-ties showed the greatest degree of suppression (ca. 98%), although the values were only marginally significantly different from those for Disrupt and NoMate, probably due to variation between plots.

**Table 1:** Trap catch of light brown apple moth and percent trap disruption following application of pheromone formulations for mating disruption over 13 weeks following treatment, expressed as covariate-adjusted, back-transformed mean summed counts, percent presence, and percent disruption (for traps at 1.5 m above ground).

Treatment	Mean count		Presence of LBAM (%)		Percent Disruption
Control	14.44	a	32.5	a	n/a
CheckMate (Suterra)	2.83	ab	10.7	ab	67.1
NoMate (Scentry)	1.25	bc	8.7	bc	73.2
Disrupt (Hercon)	0.96	bc	5.1	bc	84.3
Twist-ties (Shin-Etsu)	0.20	c	0.7	c	97.8
Splat (ISCA)	0.18	c	0.6	c	98.2

Values not sharing lower case letters are significantly different at α = 0.05 according to least significant difference tests.

**Figure 2:**
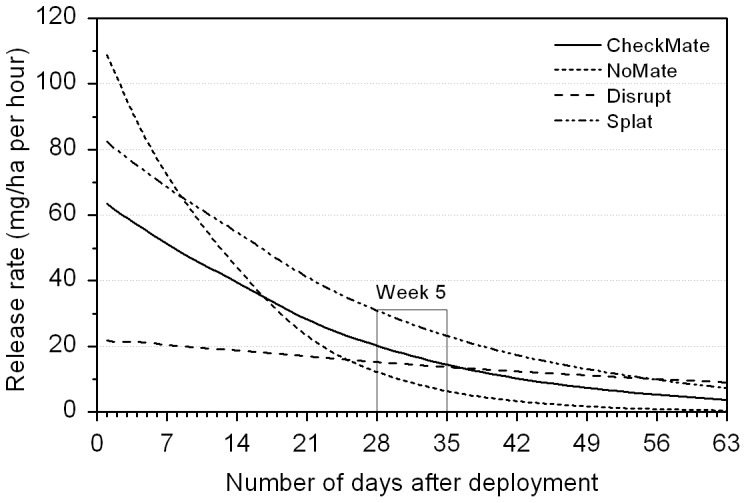
Pheromone release rates (mg ha^−1^ hr^−1^) of the main component (E-11-tetradecen-1-yl acetate) of four formulations applied for mating disruption. Release rates were calculated using actual application rates (mass/area) and the change in mass over time from decay rate curves (Fig. S4). ‘Week 5′ is highlighted as this is mentioned specifically in the results section. Values for Disrupt and CheckMate are based on the deployment target of 40 g pheromone ha^−1^. Slight under-application of NoMate and over-application of Splat, by 50%, were taken into consideration. See methods for details.

Suppression of trap catch largely mirrored the pheromone release profiles of these formulations. The small droplets of the micro-encapsulated formulations (NoMate and CheckMate) initially had the greatest rate of loss of pheromone (Fig. S4). This resulted in the highest release rates compared with the two other formulations, during the first and second weeks post-application (Fig. 2). However, by week 5, NoMate and CheckMate had already lost ca. 92% and 74%, respectively, of the main pheromone component (Fig. S4), resulting in a decreased release rate (Fig. 2). Splat and especially Disrupt had a slower and longer-lasting release, although in the case of Disrupt, this caused a considerably lower release rate already during the first few weeks following the application (Fig. 2). The actual pheromone release rate per ha (Fig. 2) also varied among treatments due to application differences among formulations. The post-trial analysis revealed that the Splat formulation exceeded the targeted application rate (discussed below).

### Effects of Trap Position (Edge Effects) and Lure Type

Edge effects were assessed by comparing trap catch and disruption relative to horizontal position within or outside treated plots, for traps near ground level (1.5 m) (Fig. S2). ANCOVAs revealed a gradient of disruption from the ‘Outside’ towards the plot centre (Table S2). This was particularly the case when traps baited with females were considered separately from traps with rubber septa lures (Table S2). A disruption gradient appeared to occur also in control plots, although it was much less pronounced (Table S2). Therefore, treatments were clearly most effective in the centre of plots, where pheromone concentrations would be expected to be higher than near or beyond the edge of the treated areas.

It is of interest to know whether *trap shutdown* is representative of *mating disruption* of females. To examine this we compared trap catch and catch suppression between traps baited with three caged freshly-emerged virgin females and those with synthetic lures (rubber septa) containing different doses of pheromone. Field survival of females was satisfactory with an average of 1.68 (±0.05 S.E.) females alive when they were replaced after one week. Traps baited with females (‘Centre - females’) had significantly higher catches, on average, than nearby traps baited with 3 mg rubber septa (‘Centre’) (Table S2). This difference was partly due to the fact that a single rubber septum was compared with several females (between 1.7 and 3.0, on average), and therefore this comparison needs to be viewed with caution. Despite this apparently greater attractiveness of female-baited traps, even these were significantly disrupted to a high degree (Table S2), indicating that the overall results are likely to be representative of successful MD of actual female moths. A dose response in trap catch or suppression relative to lure loading was not consistently apparent, although traps baited at the lowest dose appeared to have the lowest catches (Table S2).

### Vertical Transect Trap Catches and Recapture Results

Additional traps were installed along vertical transects in taller stands at ca. 1.5 m, 5 m, 9 m, 13 m and 17 m above ground to assess the vertical distribution of moths across the canopy and to determine disruption efficacy at different heights. In taller stands moths were found to be considerably more abundant in the upper canopy (at 13–17 m) than at mid-canopy and near ground level (Fig. S5). In younger stands with trees ca. 6 m tall, average trap catch at 4 m was also much higher than at 1.5 m. Analysis of covariance of vertical transects (based on 3,797 catches) indicated that traps in the upper canopy (i.e., 13–17 m in older stands; 4 m in younger stands) had significantly higher counts and presence of LBAM than those at middle or lower heights (P<0.01). In older stands, there were no significant differences in counts or presence of LBAM between the lowest height class (1.5 m) and the middle height class (5–9 m). Additional evidence for the preference of male LBAM moths for the upper canopy comes from recaptures of males that were released in plots in older stands. Of 19,190 laboratory-reared males marked with fluorescent powder that were released, 0.8% were recaptured. Ca. 68% of all recaptures were in traps at either 13 m or 17 m even though these traps represented only 19% of all the traps in these plots. Therefore, the recapture rate at 13 m and 17 m was ca. ten-fold of that recorded for the traps located at lower heights.

### Disruption of Vertical Transect Traps

**Figure 3:**
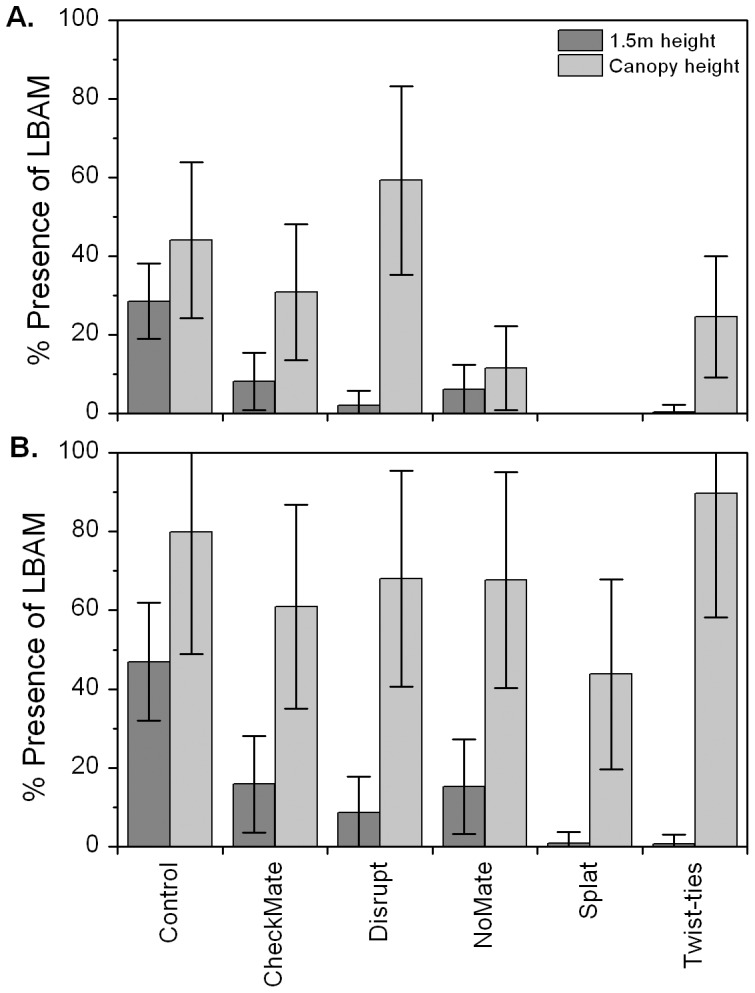
Covariate-adjusted percent presence (mean ± S.E.) of light brown apple moth in traps near ground level and at canopy height. Data shown are for weeks 1–5 (A) and for weeks 6–10 following pheromone application (B).

For the comparison of disruption efficacy at different heights across the canopy, no pre-treatment catches were available, and there were fewer traps in total in the vertical transects than for assessments of treatment effects at 1.5 m. This provided less statistical power and the results were less clear than for traps located at 1.5 m. However, the results generally indicate that control of LBAM in the upper canopy was less successful than near ground level and lasted for a shorter period of time. In weeks 1–5 post-treatment, only the Splat treatment (count, P = 0.0025; % presence, P = 0.0033) showed clear evidence of a reduction in LBAM in the upper canopy compared with the Control treatment (Fig. 3A) and, to a lesser extent, the No Mate treatment (count, P = 0.066; % presence, P = 0.098). In weeks 6–10 post-treatment, there was no evidence of any treatment differences in the upper canopy (Fig. 3B), indicating that treatments were no longer effective except near ground level. However, the presence of moths in the canopy of plots treated with Splat during weeks 6–10 (Fig. 3B) reinforces the successful disruption in the upper canopy of these plots during weeks 1–5 (Fig. 3A).

## Discussion

Our study demonstrated that aerial application of sex pheromone formulations can successfully disrupt trap catch of LBAM near ground level at a scale of 5 ha plots. Although the trap-shutdown effect was relatively short-lived for microencapsulated formulations, other formulations with a longer-lasting pheromone release provided better than 95% shutdown for at least 10 weeks near ground level. This effective period lies within the ranges reported by other studies on MD of LBAM [25], [26], [28]. Disrupt flakes, with the slowest pheromone release among the aerially applied formulations, provided an intermediate effect. The slow release of Disrupt probably resulted in lower aerial concentrations of pheromone that were insufficient for lasting disruption, but this could be improved by increasing the application rate. In addition, a rain event in the second week of this trial may have carried the Disrupt flakes to the forest floor, reducing the effectiveness of the Disrupt formulation in the upper part of the canopy.

Catches of traps baited with three females were slightly greater than catches to synthetic lures used to assess trap shutdown. The difference is probably explained by the fact that live female traps contained three females (vs. single lures). Furthermore, “centre” traps, which had the lowest catches, were surrounded by more traps than female-baited and other traps at other locations (Fig. S2). This additional competition probably contributed to the relatively lower catch rate of centre-traps. Recently, a further minor pheromone compound has been identified in the natural sex pheromone of LBAM [29], which was not included in the synthetic lures. However, even female-baited traps were effectively disrupted, indicating that the overall results are representative of successful MD of actual female moths. This was achieved despite the fact that the pheromone formulations contained at least 15% Z11-14Ac, a behavioral antagonist for LBAM. which renders release points practically unattractive if more than ca. 10% is present, relative to E-11-14Ac, the main pheromone component of LBAM [30], [31]. The lack of attraction of all formulations clearly ruled out ‘false trail following’ as a mechanism explaining trap shutdown. False trail following and competition between pheromone release devices and female moths were considered a primary mechanism according to results of a recent field cage study of another tortricid (*Cydia pomonella*) [32]. We contend that sensory impairment resulting from receptor adaptation and habituation of the central nervous system of male LBAM as well as ‘camouflage’ of calling females are more likely to be the main mechanisms [16], at least in the case of LBAM with formulations that are not attractive due to the high content of the antagonist, Z11-14Ac. A decrease in male wing fanning responsiveness to pheromone was evident with an increase in atmospheric pheromone which included the antagonist [33], suggesting that sensory adaptation was operating. This agrees with studies documenting reduced male responsiveness to the female sex pheromone, following pre-exposure of males [16], [32], [34].

The extent of trap catches in the upper canopy, demonstrated here for the first time for LBAM, strongly suggests that in arboreal situations it is critical to achieve successful MD across the vertical extent of the canopy. In taller woody vegetation, particularly when fresh growth in the understorey is limited, female LBAM are likely to occur primarily in the upper canopy, where more suitable oviposition sites are located. Achieving pheromone concentrations sufficient for successful MD at the heights of taller trees is challenging. Placing sufficient amount of formulation in the upper canopy may prove difficult due to the limited surface area available for deposition. Exposure to sunlight may accelerate degradation of formulations, and rain may drive formulations from the upper canopy. Furthermore, higher wind velocities and increased exposure to coherent turbulent structures due to the proximity of the canopy top to the atmospheric roughness sub-layer will dilute the aerial pheromone concentration [35], [36], [37]. These effects may be even more important in some LBAM-infested areas in California where trees may be taller and forest canopies more sparse compared to those in the present study. By contrast, in lower vegetation, these issues would influence MD to a lesser degree. Surprisingly few previous studies of MD in forests have examined the possible implications of the vertical dimension on pheromone concentrations.

Factors influencing plume structures from polyethylene tubing dispensers were studied in apple orchards to visualize an insect’s detection of the pheromone plume [38]. A modeling study has shown a change in biological efficacy and predicted concentration with respect to the height of orchard trees, depending on pheromone dispenser release height, canopy distribution and other factors [35]. The use of field electroantennograms was combined with a dispenser release rate model [39] and a Lagrangian model [35] to estimate atmospheric concentrations and plume structures required for disruption for LBAM in an apple orchard. Trap shutdown was achieved at an aerial concentration of >10 ng/m^3^, or emission of >5 mg per ha per hour [33]. According to our estimates based on the release rates measured in the forest, all formulations initially exceeded emissions of 5 mg per ha per hour, but only the two most effective aerially applied formulations maintained pheromone emissions at or near this level at the beginning of week 10. However, as discussed above, a greater release rate is probably required to achieve an effective aerial concentration in a forest environment. Concentrations are also influenced by the deposition of aerially-applied formulations at different heights, and this can be examined using deposition models that are suitable for forest environments [40]. With pheromone-based MD it is essential to obtain the highest aerial concentrations in the part of the canopy where the target insect is most abundant, and using modeling tools may help to achieve this objective.

Despite these complex factors, our results nevertheless show that it is possible to achieve successful MD with aerially applied formulations, and one of the formulations we tested provided suppression for many weeks at upper canopy heights and near the ground. Furthermore, the results are likely to be better near ground level *and* at greater heights when pheromone has been applied forest-wide, reducing the influence of untreated adjacent areas. However, re-application may be necessary in tall canopies and for species with an extended mating period or overlapping generations (such as LBAM in warmer climates). Evidently, to achieve successful suppression of populations in taller vegetation will require the use of tactics that can reach LBAM at these heights, and aerial application of pheromone is one of the most promising area-wide tactics available when considering environmental and human health issues.

MD is likely to be particularly successful as a tactic for eradication of recent invaders which are still of low or moderate local abundance. Such low-density populations are highly susceptible to mate-location failure causing Allee effects, a positive relationship between the size and growth rate of a population. MD raises the Allee threshold below which the population growth rate becomes negative, thereby enhancing the effect of driving the population to extinction [8], [41]. Modeling simulations showed that MD will be particularly effective when mechanisms operate like sensory impairment of males and camouflage of calling females [41], which are implicated in the case of LBAM. Therefore, MD is promising as an eradication tactic to stem the invasion of insects, although this is limited to species where a suitable pheromone is known and potentially available in large quantities.

## Materials and Methods

### Permits

All approvals and permits necessary for this work were obtained from the New Zealand Environmental Risk Management Authority (Approval numbers HSC000309– HSC000314, which included consultation of the Food Safety Authority, the Canterbury Regional Council, and the Department of Conservation). Ngai Tahu Properties (land owner) and Matariki Forest (forest manager) kindly allowed this trial to take place.

### Study Design and Plot Layout

The study took place in Eyrewell Forest (between 43°26′10.81′′S and 43°24′20.14′′S, and 172°27′14.05′′E and 172°20′30.59′′E), a *Pinus radiata* plantation forest northwest of Christchurch, New Zealand. Disruption efficacy of four aerially-applied formulations was compared with ground-applied ‘twist-tie’ polyethylene tubing dispensers (positive control) and untreated (blank) controls in a field trial using a single application over 5 ha plots (225 m×225 m) (Fig. S1, Fig. S2), replicated five times. Additional ‘external control plots’ were added as a sixth block at a greater distance from treated areas. The trap layout within each plot consisted of transects from the plot center to the plot edge and 75 m outside the plot perimeter. Traps were at least 16 m apart. Buffers of >200 m were maintained between plots and between plots, forest boundaries and open water. Plots were arranged in five blocks of spatially clustered stands of trees. A detailed canopy characterization was undertaken (Table S3). Tree heights ranged from 3–28 m.

### Pheromone Formulations

CheckMate® LBAM-F (Suterra LLC, Bend, OR) is a micro-encapsulated suspension (in water) with an average capsule size of 120 µm, with 17.6% active ingredient (i.e., LBAM pheromone). NoMate® LBAM MEC (Scentry Biologicals, Inc., Billings, MT) is a micro-encapsulated suspension (in water) with an average capsule size of 40–60 µm, with 20.3% active ingredient. Disrupt Bio-Flake® LBAM (Hercon Environmental, Emigsville, PA) is a biodegradable solid flake, each measuring approximately 3.0 mm×2.5 mm×1.9 mm and containing 13.6% of active ingredient. This formulation was applied in slurry with X3221 Micro-Tac II sticker (Lock n’ Pop, Everett, WA) with 2.5% guar gum as a suspension agent. Splat LBAM™ (ISCA Technologies, Inc., Riverside, CA) is an amorphous polymer (wax) carrier containing 10% active ingredient. CheckMate, NoMate, Disrupt, and Splat were applied aerially (details below). The pheromone composition of all these formulations was 81% E-11-tetradecen-1-yl acetate (E11-14Ac), 15% Z-11-tetradecen-1-yl acetate (Z11-14Ac), and 4% (E,E)-9,11-tetradecadien-1-yl acetate (EE9,11-14Ac) (according to supplier, Bedoukian Research, Inc., Danbury, CT).

The ground-applied ISOMATE®-LBAM PLUS is a ‘twist-tie’ polyethylene tubing dispenser (Shin-Etsu Chemical Co., Ltd., Tokyo, Japan) containing 125 mg active ingredient (68% E11-14Ac, 29% Z11-14Ac, 3% EE9,11-14Ac). These were applied to trees by hand on Feb. 19, 2008 at ca. 1.5 m above ground, at a density of ca. 600 per ha (a total of 15,000 dispensers across five plots) as a positive control [25].

### Aerial Application Details and Climatic Conditions during Application

Each of the four formulations was applied with a single aerial application using Hughes MD 500D helicopters at a target rate of 40 g LBAM pheromone per ha. The microencapsulated formulations CheckMate and NoMate were applied with rear-mounted boom systems resulting in relatively large droplets as per the target size of 390 µm. The Disrupt flake formulation was applied with a modified fertilizer bucket suspended under the helicopter. Splat was applied using a pressurized supply tank, an internal piston pump, and three oscillating solenoid valves on a rear mounted boom to give a target droplet size of 3 mm (see Table S4 for further application details). Applications occurred on Feb. 20–21, 2008. GPS tracking data from helicopters confirmed that applications of CheckMate, NoMate, and Splat were achieved with considerable accuracy in terms of targeting the plots (see also Table S4). Applications of Disrupt flakes to the specified plot area were not as accurate because of an unpredicted delay between the pump and release from the spinner both at switch on and off times. We took these delays into account as much as possible during the application by adjusting the timing of switching on and off. This ensured good plot coverage, particularly of the central area where trap disruption was assessed. Disrupt flakes initially had the slowest rate of pheromone release, until about week four (Fig. 2). The Splat application was accurately targeted to the plot area but the application rate exceeded the target rate by ca. 50% despite previously successful calibration. This apparent bias was partly compensated by the slower pheromone release compared with NoMate and CheckMate (Fig. S4). The resulting slower decay rate suggests that the aerial pheromone concentration, which is the measure ultimately relevant for successful MD, remained at an effective concentration for MD for longer. In addition, the slower decay is likely to have extended the longevity of the Splat treatment. The plot coverage of NoMate was ca. 20% below the target area but this was partly compensated by a ca. 14% greater application rate. Furthermore, the central plot area, where the main assessment of treatment effects occurred, was treated as intended.

### Trapping of LBAM

Red delta pheromone traps (Plant & Food Research, hereafter ‘PFR’), baited with 3 mg PFR lures, were installed to assess treatment effects. Lures contained a 95∶5 ratio of E11-14Ac (99.7% purity) and E,E9,11-14Ac (>99% purity) (both supplied by Pherobank, The Netherlands) loaded onto red rubber septa (Thomas Scientific Inc., Philadelphia, PA). Additional traps with 0.1 mg–3 mg PFR lures were deployed to assess dose responses and compare catches with those to three live caged female moths. We also used 3 mg and 0.1 mg rubber septa lures by Suterra (Suterra LLC, Bend, OR) for comparison (3 mg Suterra lures are used in California). Suterra lures contained a 96∶4 ratio of E11-14Ac (99.1% purity) and E,E9,11-14Ac (96.8% purity) (both supplied by Bedoukian Research). Female-baited traps contained three laboratory-reared, freshly-emerged virgin females placed in plastic vials with a mesh bottom and top to allow for pheromone dispersal. LBAM females were replaced weekly from a colony maintained by PFR.

All traps at 1.5 m were activated on Feb. 4, 2008 prior to the application of MD treatments. Additional traps in vertical transects up to 17 m high on a pulley system were set up in older stands from Feb. 17 to Mar. 4, 2008, using a motorized cherry picker. A further replicate of vertical transects was activated on Mar. 26, 2008. A total of 802 traps were used consisting of 330 transect traps placed at 1.5 m (baited with 3 mg PFR lures), 180 dose response traps at 1.5 m (30 each of 0.1 mg, 0.3 mg, 1 mg, 3 mg PFR lures and 0.1 mg and 3 mg Suterra lures), 120 female-baited traps at 1.5 m (four per plot), 154 additional traps in tall vertical transects (up to 17 m height, in tall stands, 3 transects per plot, 2 replicates; all baited with 3 mg PFR lures), and 18 additional traps in short vertical transects (up to 4 m height, in young stands, 3 transects per plot, all baited with 3 mg PFR lures). Traps were checked and cleared on the day when treatments were applied and then at weekly intervals until May 15–16, 2008. A final collection was made on June 12–13, 2008.

### Releases of Male LBAM

Between Mar. 6 and May 2, 2008, a total of 19,190 laboratory-reared male LBAM, marked with fluorescent powder, were released to supplement populations in older stands to assist with the detection of MD effects. Weekly releases of between 100 and 480 moths per plot were made at plot centers. Numbers varied between weeks, depending on moth availability. From March onwards, all trapped moths were viewed under UV light to detect recaptures of dyed moths. Additional releases and recaptures after 2 May were excluded from the recapture analysis because few catches occurred after the onset of the southern hemisphere winter.

### Longevity of Formulations

Pheromone release over time was assessed in an untreated part of the forest. Twenty-five 3 µl droplets of CheckMate, NoMate and Splat, and 25 Disrupt flakes, were applied to 5×10 cm Strathmore canvas paper and placed in a shaded area, protected by wire mesh. Three replicates were removed for analysis on days 0 (Feb. 23, 2008), 1, 3, 7, and then in weekly intervals until day 63. Each card was placed in a borosilicate glass vial and held in a freezer or under dry ice. LBAM pheromone was extracted with 25 ml of acetone/hexane (1/1) and analyzed using an Agilent 6890N Network GC system (Agilent Technologies 7683 B Series auto injector; Agilent 19091 J-413, HP-5 5% Phenyl Methyl Siloxane column (30 m×320 µm×0.25 µm; 100DISP1, splitless, 100°C (2 min), 15°C/min to 280°C (10 min); Hydrogen carrier). Loss of mass of the main active ingredient (E11-14Ac) over time was determined using these GC measurements. For liquid and waxy formulations, mass for the initial day was calculated using the specific gravity of E11-14Ac. Using the day zero and subsequent E11-14Ac mass measurements, an exponential decay curve was fitted to the data. To compare treatment decay rates, mass from the sampling days was normalized to the day zero mass. Decay curves were also used to estimate the resulting pheromone release rates per ha for given application rates of formulations.

### Data Analysis

Two response variables were analyzed, (i) trap catch (‘count’) per hundred trap days (log transformed using ln(N+0.1)) and (ii) the percentage of traps catching LBAM within each plot (analyzed using angular or arcsine transformation). The main analysis was for data from traps at 1.5 m height. Analyses of covariance using SAS Version 9.1 PROC MIXED were used to test for treatment differences. The log-transformed trap count for each plot in the 2 weeks prior to treatment was used as a covariate. Separate analyses of covariance were performed for each response variable using plot means for each assessment (weekly), for five-weekly averages (Weeks 1–5 and 6–10), and for the average across all 13 weeks following treatment, along with least significant difference post-hoc tests comparing all treatments. Traps located ‘outside’ plot areas (75 m from the edge of the treated area) were excluded from this analysis. All control traps were included in the analysis. Percentage disruption was calculated as 100-(100*(treatment catch/control catch)).

Analyses comparing trap position (Outside, Edge, Centre/Edge, Centre) and lure type (rubber septa vs. females) within each plot were performed with ANOVAs separately for control plots and for treated plots combined. These were carried out using PROC MIXED with plot included in the model as a random effect and position × type as a fixed interaction effect.

For vertical transects, no pre-treatment catches were available above 1.5 m height, and pre-treatment log count at 1.5 m height was therefore used as a covariate. Count and presence data was classified into 3 height classes, (1) 1.5 m, (2) middle (5–9 m in older stands only), (3) upper canopy (13–17 m in older stands or 4 m in younger stands). Average count and presence by height class and plot (excluding outside traps) were then subjected to analysis of covariance (ANCOVA) using PROC MIXED with fixed-effect model terms for treatment, height class, and the interaction between these two factors, and random plot effects.

## Supporting Information

Figure S1 **Aerial view of mating disruption plot locations in Eyrewell Forest (New Zealand).**
(TIF)

Figure S2 **Plot layout showing pheromone aerial treatment area and core area, trap locations and lures used.** Most lures were standard Plant&Food Research (‘PFR’) lures loaded with 3 mg LBAM pheromone. Other lures were used to examine dose responses and to compare PFR and Suterra (‘S’) lures which are used in California. See text for more details on plot design and lure types.
(TIF)

Figure S3 **Effects of application of pheromone formulations on trap catch (percent presence) of light brown apple moth.** Note: Results are for traps at 1.5 m above ground shown as back-transformed covariate-adjusted percent moth presence in traps. Data for weeks 11–13 are based on very low catches at the end of the flight period.
(TIF)

Figure S4 **Loss of pheromone in the field expressed as a percentage of the main component (E-11-tetradecen-1-yl acetate) remaining over time for each formulation, with 95% confidence intervals, based on analysis by gas chromatography of extracts from 3 µl droplets or Disrupt ‘flakes’ applied to canvas paper cards placed in the forest where the trial took place.** See methods for details. Note that the actual aerial application of Splat resulted in a range of droplet sizes, with 3 µl being at the lower range of Splat droplets. A proportion of Splat droplets were considerably larger than 3 µl, and the actual longevity of the Splat treatment was therefore somewhat longer than shown here.
(TIF)

Figure S5 **Vertical distribution of catches of light brown apple moth (mean ± S.E.) in tall stands based on traps in control plots and untreated areas (n = 22 transects for all heights except for 17 m where n = 11).**
(TIF)

Table S1 **Analysis of covariance (ANCOVA) table showing significance of covariate and treatment effects in the analysis of log-transformed counts and angular-transformed percent presence of light brown apple moth for the average of the 13 weeks following treatment, and for weeks 1–5 and 6–10 following treatment, and each of the 13 weeks following treatment.**
(DOCX)

Table S2 **Trap catch of male light brown apple moth at different locations within and outside plots and for different lure types following application of pheromone formulations.** Covariate-adjusted, back-transformed mean summed counts and percent presence are shown for control plots and for all treated plots combined over 13 weeks following application, for traps at 1.5 m above ground. Values (within columns) not sharing lower case letters are significantly different at α = 0.05 according to least significant difference tests.
(DOCX)

Table S3 **Mean tree height, canopy thickness (i.e., green crown height) and tree density for replicated plot areas (n = 5).** Block 6 contained five extra control plots at a greater distance from treated areas.
(DOCX)

Table S4 **Target swath width, speed, altitude of application (above ground) and droplet size for the four helicopter-applied formulations.** See footnote ^§^for further application details.
(DOCX)
