# Supplementary figures and images for: Aerial Application of Pheromones for Mating Disruption of an Invasive Moth as a Potential Eradication Tool
Source: PLoS One. 2012 Aug 24;7(8):e43767. doi: 10.1371/journal.pone.0043767 (PMC3427152; doi:10.1371/journal.pone.0043767)

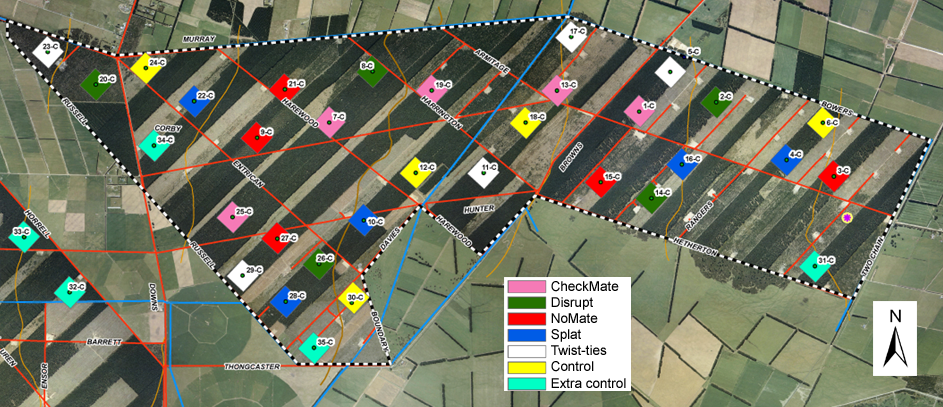

Supplement: Figure S1 — Aerial view of mating disruption plot locations in Eyrewell Forest (New Zealand). (TIF) [file pone.0043767.s001.tif]

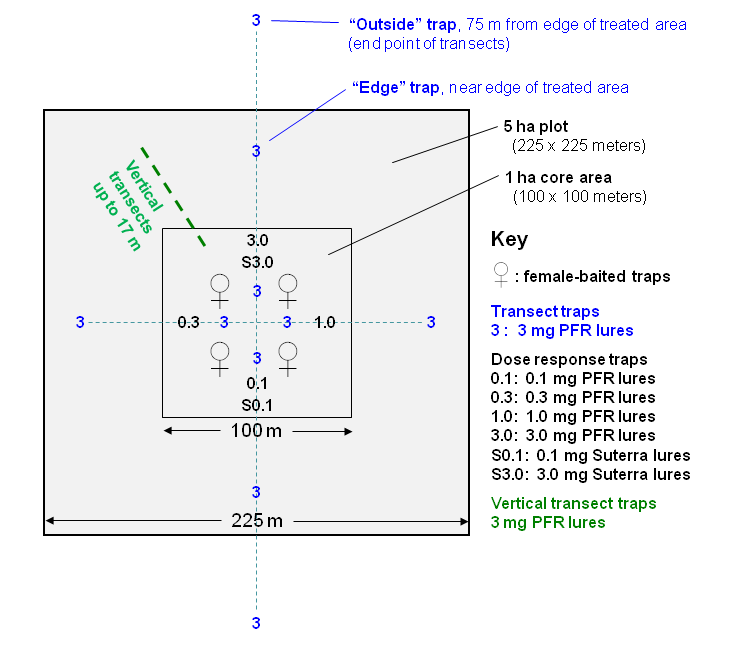

Supplement: Figure S2 — Plot layout showing pheromone aerial treatment area and core area, trap locations and lures used. Most lures were standard Plant&Food Research (‘PFR’) lures loaded with 3 mg LBAM pheromone. Other lures were used to examine dose responses and to compare PFR and Suterra (‘S’) lures which are used in California. See text for more details on plot design and lure types. (TIF) [file pone.0043767.s002.tif]
