# Supplementary material for: Aerial Application of Pheromones for Mating Disruption of an Invasive Moth as a Potential Eradication Tool
Source: PLoS One. 2012 Aug 24;7(8):e43767. doi: 10.1371/journal.pone.0043767 (PMC3427152; doi:10.1371/journal.pone.0043767)
Supplement: Table S1 — Analysis of covariance (ANCOVA) table showing significance of covariate and treatment effects in the analysis of log-transformed counts and angular-transformed percent presence of light brown apple moth for the average of the 13 weeks following treatment, and for weeks 1–5 and 6–10 following treatment, and each of the 13 weeks following treatment. (DOCX) [file pone.0043767.s006.docx]

**Table S1**. **Analysis of covariance (ANCOVA) table showing significance of covariate and treatment effects** in the analysis of log-transformed counts and angular-transformed percent presence of light brown apple moth for the average of the 13 weeks following treatment, and for weeks 1-5 and 6-10 following treatment, and each of the 13 weeks following treatment.

|  | Counts | | | | Presence/Absence | | | |
| --- | --- | --- | --- | --- | --- | --- | --- | --- |
| Week | Covariate | | Treatment | | Covariate | | Treatment | |
|  | d.f. 1,28 | | d.f. 5,28 | | d.f. 1,28 | | d.f. 5,28 | |
|  | F-value | Prob>F | F-value | Prob>F | F-value | Prob>F | F-value | Prob>F |
| All weeks combined (weeks 1-13) | 28.14 | <.0001 | 5.81 | 0.0008 | 33.09 | <.0001 | 8.08 | <.0001 |
| Weeks 1-5 | 26.63 | <.0001 | 5.40 | 0.0013 | 39.26 | <.0001 | 7.18 | 0.0002 |
| Weeks 6-10 | 20.45 | <.0001 | 6.83 | 0.0003 | 23.52 | <.0001 | 7.09 | 0.0002 |
|  |  |  |  |  |  |  |  |  |
| 1 | 33.71 | <.0001 | 11.78 | <.0001 | 38.84 | <.0001 | 9.16 | <.0001 |
| 2 | 37.84 | <.0001 | 4.63 | 0.0033 | 49.04 | <.0001 | 4.70 | 0.0031 |
| 3 | 29.95 | <.0001 | 3.81 | 0.0093 | 27.97 | <.0001 | 3.69 | 0.011 |
| 4 | 32.12 | <.0001 | 3.46 | 0.015 | 33.50 | <.0001 | 3.62 | 0.012 |
| 5 | 23.65 | <.0001 | 2.99 | 0.028 | 21.38 | <.0001 | 2.93 | 0.030 |
| 6 | 32.24 | <.0001 | 3.73 | 0.010 | 26.28 | <.0001 | 3.15 | 0.022 |
| 7 | 21.95 | <.0001 | 6.71 | 0.0003 | 26.31 | <.0001 | 6.99 | 0.0002 |
| 8 | 21.30 | <.0001 | 5.01 | 0.0021 | 24.74 | <.0001 | 5.18 | 0.0017 |
| 9 | 13.62 | 0.0010 | 6.62 | 0.0003 | 8.65 | 0.0010 | 5.49 | 0.0012 |
| 10 | 12.25 | 0.0016 | 4.65 | 0.0032 | 19.88 | 0.0016 | 5.87 | 0.0008 |
| 11 | 15.52 | 0.0005 | 2.75 | 0.038 | 13.68 | 0.0005 | 2.32 | 0.070 |
| 12 | 15.01 | 0.0006 | 1.80 | 0.14 | 21.65 | 0.0006 | 1.97 | 0.11 |
| 13 | 12.78 | 0.0013 | 2.88 | 0.032 | 10.27 | 0.0013 | 2.54 | 0.051 |
