# Supplementary material for: Aerial Application of Pheromones for Mating Disruption of an Invasive Moth as a Potential Eradication Tool
Source: PLoS One. 2012 Aug 24;7(8):e43767. doi: 10.1371/journal.pone.0043767 (PMC3427152; doi:10.1371/journal.pone.0043767)
Supplement: Table S2 — Trap catch of male light brown apple moth at different locations within and outside plots and for different lure types following application of pheromone formulations. Covariate-adjusted, back-transformed mean summed counts and percent presence are shown for control plots and for all treated plots combined over 13 weeks following application, for traps at 1.5 m above ground. Values (within columns) not sharing lower case letters are significantly different at α = 0.05 according to least significant difference tests. (DOCX) [file pone.0043767.s007.docx]

**Table S2**. **Trap catch of male light brown apple moth at different locations within and outside plots and for different lure types** following application of pheromone formulations. Covariate-adjusted, back-transformed mean summed counts and percent presence are shown for control plots and for all treated plots combined over 13 weeks following application, for traps at 1.5 m above ground. Values (within columns) not sharing lower case letters are significantly different at α = 0.05 according to least significant difference tests.

| Trap type and location within plot | Mean count | | | | Presence of LBAM (%) | | | |
| --- | --- | --- | --- | --- | --- | --- | --- | --- |
|  | Control mean | | Treated mean | | Control mean | | Treated mean | |
| Centre (3 mg lures) | 4.81 | c | 0.29 | d | 22.7 | c | 1.4 | c |
| Centre - females (caged female LBAM) | 45.60 | a | 0.73 | bc | 53.8 | a | 5.5 | b |
| Centre/Edge (0.1 - 3 mg dose-response lures) | 9.78 | bc | 0.57 | cd | 30.1 | bc | 3.4 | bc |
| Edge (3 mg lures) | 26.58 | ab | 1.46 | b | 45.3 | ab | 7.6 | b |
| Outside (3 mg lures) | 29.67 | a | 9.30 | a | 51.9 | a | 31.2 | a |
