# Supplementary material for: Aerial Application of Pheromones for Mating Disruption of an Invasive Moth as a Potential Eradication Tool
Source: PLoS One. 2012 Aug 24;7(8):e43767. doi: 10.1371/journal.pone.0043767 (PMC3427152; doi:10.1371/journal.pone.0043767)
Supplement: Table S3 — Mean tree height, canopy thickness (i.e., green crown height) and tree density for replicated plot areas (n = 5). Block 6 contained five extra control plots at a greater distance from treated areas. (DOCX) [file pone.0043767.s008.docx]

**Table S3.** **Mean tree height, canopy thickness (i.e., green crown height) and tree density** for replicated plot areas (n = 5). Block 6 contained five extra control plots at a greater distance from treated areas.

| Block | Mean tree height (m) | Live canopy thickness (m) | Mean stems per ha of *P. radiata* trees | Mean stems per ha of regenerating seedlings |
| --- | --- | --- | --- | --- |
| 1 | 24.9 | 13.3 | 350 | N/A |
| 2 | 17.6 | 11.2 | 583 | N/A |
| 3 | 4.5 | 4.5 | 558 | 1614 |
| 4 | 5.9 | 5.9 | 578 | 1366 |
| 5 | 13.4 | 10.9 | 546 | 2375 |
| 6 | 10.7 | 8.7 | 558 | 1401 |
