# Supplementary material for: Aerial Application of Pheromones for Mating Disruption of an Invasive Moth as a Potential Eradication Tool
Source: PLoS One. 2012 Aug 24;7(8):e43767. doi: 10.1371/journal.pone.0043767 (PMC3427152; doi:10.1371/journal.pone.0043767)
Supplement: Table S4 — Target swath width, speed, altitude of application (above ground) and droplet size for the four helicopter-applied formulations. See footnote §for further application details. (DOCX) [file pone.0043767.s009.docx]

**Table S4. Target swath width, speed, altitude of application (above ground) and droplet size for the four helicopter-applied formulations**. See footnote ^§^ for further application details.

|  | Disrupt | Splat | NoMate | CheckMate |
| --- | --- | --- | --- | --- |
| Swath width (m) | 18 | 14 | 12 | 12 |
| Speed (knots) | 45 | 30 | 45 | 45 |
| Altitude (m) | 43 | 43 | 43 | 43 |
| Droplet or flake size (μm) | 3000 | >5000 | 390 | 390 |

^§^ Each of the four formulations was applied with a single aerial application using Hughes MD 500D Helicopters with 5 rotor blades (8.05 m rotor diameter) at a target rate of 40 g LBAM pheromone per ha. The microencapsulated formulations CheckMate (Suterra) and NoMate (Scentry) were applied with a rear-mounted boom spray system with hydraulic recirculation agitation, boom pressure 30 psi and three Turbo TeeJet wide angle flat spray nozzles # 11005 (Spray Systems Co., Wheaton, Illinois), resulting in droplets close to the target size of ca. 390 μm. The Disrupt flake formulation (Hercon) was applied with a modified fertilizer bucket suspended under the helicopter by a 6 m sling. Two 24 volt peristaltic pumps were used to control slurry output with a multi-vane disc spinning dispenser. Splat (ISCA) was applied using three solenoid valves at the center and 3.1 m to the left and right along a rear mounted boom. A converted beer keg, pressurized to 7 psi by an internal piston pump, was used as supply tank, with 25 mm high pressure hoses leading to the solenoid valves, which were pulsed at approximately 4.5 per second to give a target droplet size of ca. 3 mm. Swath widths and application speed were set according to the requirements for the different formulations. Calibration to the target rates was achieved for all formulations although the actual application rates ranged from ca. 40 to 60 g pheromone per ha. To ensure the droplets and flakes reached their target during the application, we set conservative limits on the spraying conditions (i.e., wind speed < 10 km/h, humidity > 70%, temperature < 20°C). Wind speed, temperature and humidity were monitored using meteorological stations set up in the forest. Suitable conditions for accurate application occurred mainly during morning and evening. Conditions were considered less stringent for Disrupt flakes which are less susceptible to evaporation and drift due to their comparatively large size. Aerial applications occurred on 20 and 21 February 2008.
